# Supplementary material for: Individual differences and the transposed letter effect during reading
Source: PLoS One. 2024 Feb 28;19(2):e0298351. doi: 10.1371/journal.pone.0298351 (PMC10901340; doi:10.1371/journal.pone.0298351)
Supplement: Attachment — Submitted filename: Response to Reviewers.docx [file pone.0298351.s002.docx]

Dear Prof. Comesaña Vila,

My co-authors and I would like to thank you for your comments on previous versions of this manuscript. We were happy to make the changes that you have suggested. Below we provide some additional information to address the comments. We have also highlighted changes made in the revised manuscript.

We look forward to hearing from you soon.

Kind regards,

Charlotte Lee

PONE-D-23-23508R1

Individual differences and the transposed letter effect during reading

PLOS ONE


**Editor Comments:**

Thank you again for submitting your manuscript ID PONE-D-23-23508R1 entitled "Individual differences and the transposed letter effect during reading " to Plos One. Your manuscript has been reviewed again by myself and i truly think you have adressed the suggestions made by the two reviewers and improved the manuscript. Thus, I am very happy to accept it with minor revision. Below you could find my suggestions.

In the abstract please make explicit what the acronym PCA stands for (Principal Component Analysis)

**We have now clarified that the acronym PCA refers to a principal components analysis in the abstract on line 33-34.**

Line 139, please replace “Reading studies” with “Sentence-reading studies”

**We have now made this change on line 140.**

Line 168, remove the comma in Gómez et al., [50]

**This has now been removed on line 169.**

Lines 173-175, you stated that lexical decision times were modulated by individual differences in reading ability but then you say that “negligible differences were associated with word-reading”. When you say “associated with word-reading” did you mean associated with the RTs found in the lexical decision task or using other tasks with other non-experimental words? Re-write this in order to be clearer.

**We have now included clarification on lines 174-175 that word reading skill was measured by a subtest from PROLEC-R [54], rather than during the lexical decision task.**

Line 177, when you talk about the principal componente analysis here put the acronym between parentheses.

**The acronym (PCA) has now been included on line 179.**

Line 283 please makes explicit what the acronym RAN stands for (Rapid Automatized Naming)

**This now reads “Rapid Automatized Naming (RAN) scores” on line 284.**

Line 295 an space after the = sign is needed

**This has now been added on line 296.**

Lines 336-338 please review the examples you gave because there are no transposed letters at all except for the internal letters:
(at the beginning e.g.,problem, or end e.g., problem, of a word) than internal letters (e.g., problem/probelm) in a sentence reading study.

**The relevant letter transpositions have now been corrected in the examples on lines 338 and 339. We thank you for highlighting this issue.**

Lines 668-679. Please include what i have written below in uppercase.

“…used by Pagán et al., [49] when studying children’s eye movements (we thank an anonymous reviewer for this suggestion). Another way to extend this research would be to investigate the flexibility of letter position encoding in second language learners, who are simultaneously highly skilled L1 readers and developing L2 reader (we thank a second anonymous reviewer for this suggestion). Individual differences in reading
skills in both languages may be included in such investigations, to address
whether L1 and L2 reading skills (e.g., vocabulary size, spelling ability and
reading experience) influence letter position encoding when reading in L2. IT IS WORTH NOTING HERE THAT a recent study [91] investigated transposed letter effects in native Chinese speakers learning English as a second language and found that L2 vocabulary knowledge (measured by the LexTALE) did not modulate the flexibility of readers’ letter position encoding in English (L2).”

**This wording has now been included on lines 676 – 677.**

Lines 721-725, you stated that “the comprehension subtest of the
WIAT-II was associated with lexical proficiency (PC1) whereas the
comprehension subtest of the NDRT was distinct and that these tests were
weakly correlated (r = 0.15). Although both are standardised measures of
comprehension, it appears that they measure distinct underlying constructs”.

Could you speculate about the construct thai is measured by the NDRT?

**Thank you for this comment. Although we are a bit hesitant to speculate on the construct measured by the NDRT comprehension test from our data, we have now provided a suggestion (on lines 727 – 730) from previous research by Coleman et al (2010) and Ready et al (2013) that the NDRT may be closely associated with IQ. Relevant references are now included on lines 1008 – 1013.**

**Ready RE, Chaudhry MF, Schatz KC, Strazzullo S. "Passageless" administration of the Nelson-Denny reading comprehension test: Associations with IQ and reading skills. J Learn Disabil. 2013;46(4):377-384. doi:10.1177/0022219412468160**

**Coleman C, Lindstrom J, Nelson J, Lindstrom W, Gregg KN. Passageless comprehension on the Nelson-Denny reading test: well above chance for university students. J Learn Disabil. 2010;43(3):244-9. doi:10.1177/0022219409345017.**
